# Exploring the Role of a Cytokinin-Activating Enzyme LONELY GUY in Unicellular Microalga *Chlorella variabilis*

**DOI:** 10.3389/fpls.2020.611871

**Published:** 2021-01-29

**Authors:** Saraswati Nayar

**Affiliations:** Division of Plant Molecular Biology, Rajiv Gandhi Centre for Biotechnology, Thiruvananthapuram, India

**Keywords:** algae, biomass, cell cycle, *Chlorella*, cytokinin activation, LONELY GUY, photosynthesis, non-seed plants

## Abstract

LONELY GUY has been previously characterized in flowering plants to be involved in the direct activation of cytokinins. In this study, the function of the only *LONELY GUY gene* (*CvarLOG1*) from unicellular green microalga *Chlorella variabilis* NC64A has been investigated. *CvarLOG1* expressed mainly in the lag and log phases of growth and was confirmed to be a cytokinin-activating enzyme. Overexpression of *CvarLOG1* in *Chlorella* led to extended life in culture by almost 10–20 days, creating a “stay-green” phenotype. In the transformed alga, the cell cycle was lengthened due to delayed entry into the G2/M phase contrary to the known role of cytokinins in stimulating G2/M transition possibly due to excessive levels of this hormone. However, due to the sustained growth and delayed senescence, there was an increase in cell number by 11% and in biomass by 46% at the stationary phase, indicating a potential application for the biofuel industry. The total carbohydrate and lipid yield increased by approximately 30 and 20%, respectively. RNA-Seq-based transcriptomic analysis revealed that the genes associated with light and dark reactions of photosynthesis were upregulated, which may be the reason for the increased biomass. These data show that *LOG* plays an essential role during the cell cycle and in the functioning of the chloroplast and that the pathway leading to direct activation of cytokinins *via* LOG is functional in algae.

## Introduction

The presence and role of active hormones had been long speculated in algae, but the first proof of the bioactive forms of auxin, cytokinin, Gibberellic Acid (GA), brassinosteroid, Abscisic Acid (ABA), Jasmonic Acid (JA), and polyamines came from the work of Tarakhovskaya et al. (2007). Later, more data describing the exact levels for auxin, cytokinin, GA, brassinosteroid, and ABA were added for the microalgae (Tarakhovskaya et al., 2007; Stirk et al., 2013a, b, 2014); however, their exact function remained unknown. In 2010, the *Chlorella variabilis* NC64A genome was decoded (Blanc et al., 2010), and the presence of various classes of hormonal genes was discovered in this microalga, including those involved in cytokinin biosynthesis and signaling (Blanc et al., 2010). Cytokinins are a class of adenine-derived small-molecule phytohormones that play a vital role in many facets of plant growth and development, such as cell division, cell growth, and differentiation through the interplay with auxins (Howell et al., 2003; Ferreira and Kieber, 2005; Sakakibara, 2006). Discovery of these genes in *Chlorella* raises questions about the role of cytokinins in the development of these microalgae. In comparison to the vascular plants, microalgae lack specific proteins involved in hormone signaling; thus, the exact physiological role of cytokinins in the microalgae remains to be explored (Lu and Xu, 2015). Stirk et al. (2013b) looked at the levels of cytokinin in different strains of microalgae and found that different cytokinins accumulated in consistent profiles, i.e., *c*Z > iP > *t*Z > DHZ in all the actively dividing strains (Stirk et al., 2013b). In microalgae, it had been speculated that the levels of active cytokinins are controlled by *de novo* biosynthesis (Sakakibara, 2006). In a later study, this speculation was proved to be accurate as precursors of the *de novo* biosynthesis were found to be high in most of the microalgal strains analyzed (Stirk et al., 2013b). Furthermore, components of microalgal phytohormone biosynthetic pathways were found to share a resemblance to that of higher plants (Lu and Xu, 2015), whereas hormone signaling pathways seem to be significantly different. Most of the studies related to the functional roles of hormones in microalgae are derived from studying the direct application of higher plant hormones on microalgal cells. To date, little is known about the role of hormone-related genes in microalgae (Lu and Xu, 2015). Taken together, these studies point toward certain equivalence between hormone biosynthetic pathways in microalgae and higher plants, whereas the evidence related to the roles these perform is largely missing. Therefore, further studies on the functional role of phytohormones in microalgal systems would be necessary to understand their full potential and put this knowledge into any biotechnological use (Lu and Xu, 2015).

In this study, the role of an algal ortholog of a cytokinin biosynthesis gene *LONELY GUY (LOG)* has been explored. Cytokinin riboside 5′-monophosphate phosphoribohydrolase (LONELY GUY) catalyzes the release of free-base, active cytokinin species from cytokinin ribotides in a single enzymatic step, which was first discovered in rice and then in *Arabidopsis* (Kurakawa et al., 2007; Kuroha et al., 2009). This enzyme hydrolyzes only cytokinin riboside 5′-monophosphate but not AMP, suggesting that it is specifically involved in cytokinin activation (Kurakawa et al., 2007; Kuroha et al., 2009). This enzyme has also been characterized in cytokinin activation in other plants such as *Medicago truncatula* and *Solanum lycopersicum*, and in certain microbes such as *Mycobacterium*, *Corynebacterium*, and *Claviceps purpurea* (Eviatar-Ribak et al., 2013; Mortier et al., 2014; Naseem et al., 2015; Samanovic et al., 2015; Seo et al., 2016). It is known that micro-algae are a good source of feedstock for the biofuel industry (Stephenson et al., 2011), but there is a need for strain improvement to cut production costs (Sharma et al., 2018). Therefore, for strain improvement, the possibility of increasing biomass by overexpressing the cytokinin-activating gene *LONELY GUY (LOG)* in *Chlorella variabilis* was explored as previous studies show that exogenous application of cytokinin in microalgae leads to increase in growth rate (Lu et al., 2014).

In this report, the functional characterization of a LOG ortholog in *Chlorella variabilis* has been undertaken. This study covers the phylogenetic analysis of CvarLOG1 protein, mRNA expression, *in vivo* localization of the protein, biochemical characterization of the recombinant enzyme, overexpression study for strain improvement, and discovery of downstream pathways of this enzyme using RNA sequencing of the overexpressor. This study will, therefore, pave the way for strain development for better biofuel feedstock using native hormone-related genes.

## Materials and Methods

### Microalgal Cultures and Growth Conditions

Cultures of *Chlorella variabilis* NC64A wild type strain (Accession Number: CCAP 211/84) was purchased from Culture Collection of Algae and Protozoa (CCAP), Scotland. Wild type and overexpression cells were cultured in a 250-ml Erlenmeyer flask containing 80 ml of modified Bold’s basal medium (MBBM) at 25°C in a 12:12 h/L:D cycle. The cultures (wild type and LOG OX) were manually agitated every day. Growth kinetics of the cultures was examined by measuring optical density at 750 nm (OD_750_) with a spectrophotometer (Shimadzu, Japan) for three biological replicates each.

### Phylogenetic Analysis

Complete amino acid sequences of LOG proteins having over 50% identity to AtLOG3 were identified by BLASTp search in *Populus trichocarpa*, *Selaginella*, and selected algae (*Chlorella variabilis*, *Volvox*, *Chlamydomonas*, *Coccomyxa subellipsoidea*). The LOG protein sequences for *Physcomitrella*, Rice, and *Arabidopsis* were taken from a previous study (Kuroha et al., 2009). The sequences from the non-seed plants and seed plants were aligned using the default setting of ClustalW (Thompson et al., 1994). A phylogenetic tree was constructed in the MEGA 7 program^1^ using the default setting of the neighbor-joining method (Saitou and Nei, 1987). Bootstrap analysis was conducted with 1,000 replicates.

### Cloning of LOG cDNA

The *CvarLOG1* gene identified in *Chlorella variabilis* NC64A was amplified using CDS-specific primers (see Supplementary Table 5) using Phusion polymerase as per the manufacturer’s protocol (New England Bio labs, United States) from log phase cDNA and cloned in pENTR/D-TOPO using the TOPO cloning kit (Invitrogen, United States) according to the manufacturer’s protocol. From the entry vector pENTR/D-TOPO, it was cloned into overexpression vector pMDC32 (Curtis and Grossniklaus, 2003) and localization vector pDH51-YFP (Zhong et al., 2008) using LR clonase cloning kit (Invitrogen, United States) as per manufacturer’s protocol.

### Intracellular Localization

Particle bombardment was carried out using GenePro 2000 He particle delivery system (ENDEAVOUR Enterprises, Bio-Mech Engineering Services, Hyderabad) with LOG-YFP and YFP only constructs in onion peel cells according to the protocol described in an earlier study (Nayar et al., 2014). For each construct, 2.5 μg of DNA was used to coat 1.5 mg (0.5 mg per shot) 1 μm tungsten particles, and the following shooting parameters were used: 27 mm Hg vacuum, 1,100 psi helium pressure, and target distance of 9 cm. The plates were incubated for 16 h at 25°C, in the dark. The onion peels were observed for YFP under the confocal microscope using the 488-nm laser for excitation (Nikon, Japan). The bright field as well as fluorescence images were taken separately and overlaid by using Adobe^®^ Photoshop CC. This experiment was repeated three times to test the repeatability.

### LOG Enzyme Expression and Purification

The coding region from Cvar*LOG1* was amplified from *Chlorella variabilis* cDNA. The primers used for the PCR were LOG pET28 F and LOG pET28 R (see Supplementary Table 5). The PCR products were ligated into the pET28a (Novagen) to express His-tagged recombinant proteins. The BL21 (DE3) strain was used to express the recombinant protein that was induced in LB broth with 1 mM isopropyl-D-thiogalactopyranoside for 5 h at 37°C. After induction, *Escherichia coli* cells were pelleted and stored at −20°C until further use. The protein was extracted using the Capturem His-Tagged Purification Miniprep Kit (Takara Bio, Japan) to purify recombinant proteins according to the manufacturer’s protocol.

### Enzyme Assay

Enzyme activity of LOG as a cytokinin nucleoside 5′-monophosphate phosphoribohydrolase was measured by a method described in an earlier study (Kurakawa et al., 2007) with minor modifications. For conversion analysis, recombinant protein activity was measured by incubating 6 μg of recombinant LOG in a reaction mixture (50 mM Tris, 1 mM MgCl2, 1 mM dithiothreitol, pH 7) with 20 μM substrate (iPRMP or tZRMP), at 30°C for 2 h. The reaction was stopped with three volumes of acetone and stored at −80°C for 30 min. Insoluble material was removed by centrifugation at 15,000 × *g* for 15 min, at 4°C, and the supernatant was dried using a speed vac (Labconco Corp., MO, United States) at 45°C. The resulting material was dissolved in 1% acetic acid. Cytokinins were separated using UHPLC (Waters) on a Acquity BEH C18 column (1.7 μm, 2.1 × 100 mm, Waters) at a flow rate of 0.3 ml min^–1^ with gradient of solvent A (1% acetic acid) and solvent B (acetonitrile) according to the following gradient profile: 0 min, 99% A + 1% B; 1 min, 99% A + 1% B; 1.2 min, 93% A + 7% B; 4 min, 90% A + 10% B; 11 min, 60% A + 40% B; 13.50 min, 50% A + 50% B, 15 min 99% A + 1% B. The column temperature was maintained at 40°C, and samples were maintained at 8°C. This experiment had two replicates each and was repeated three times to check repeatability. Cytokinins were separated and monitored by their absorbance at a wavelength of 270 nm. Samples and standards were dissolved in 1% acetic acid. For the determination of kinetic parameters of CvarLOG1 for iPRMP and *t*ZRMP substrates, 4 μg of recombinant LOG was used with various concentrations of substrates. GraphPad Prism was used to calculate the kinetics.

### Transformation

Transformation of *Chlorella variabilis* was done according to a method described previously with slight modifications, which yielded 22 independent LOG OX lines (Niu et al., 2011). The transformation was done three times to confirm the phenotype. *Chlorella* cells in the exponential phase were collected by centrifuge at 1,350 × *g* for 10 min. A pellet containing the equivalent of 3 × 10^6^ *Chlorella* cells was resuspended with 150 μl of 1.0 mol/L NaCl, then mixed with 150 μl (0.1 mol/L mannitol), and kept on ice for 30 min. Suspension aliquots of 0.3 ml in volume were mixed with 0.5 μg plasmid pMDC32-LOG and then transferred into an electroporation cuvette (0.2 cm gap, Sigma). After electroporation, the cells were transferred into 10 ml of MBBM medium, kept in the dark for 2 h, then incubated at 25°C for 24 h (12L:12D). Later, the cells were collected by centrifugation at 1,500 × *g* for 5 min and resuspended in 1 ml of MBBM medium. The cells were finally spread on the MBBM selection medium supplemented with 15 g/L of agar containing hygromycin (5 mg/L). Five independent overexpression lines were maintained on solid medium for 64 generations without any loss of phenotype and are still being maintained.

### Algae Cell Disruption and Colony PCR

Algae crude extract was prepared, and colony PCR was done as described in an earlier study (Radha et al., 2013). Colonies were chosen from wild type and seven LOG OX lines and collected in a microcentrifuge tube individually. To each colony, about 20 mg of autoclaved powdered glass was added and ground using a sterile micropestle. Cells were resuspended in 100 μl of Tris-ethylenediaminetetraacetic acid (EDTA) buffer (pH 8.0) and centrifuged at 10,000 rpm for 5 min, and the supernatant was collected in a fresh tube. One microliter of the supernatant was used for colony PCR. Colony PCR was carried out using the supernatant by High Fidelity Phusion Polymerase (New England Bio Labs, United States) with hygromycin-specific primers (Supplementary Table 5) as per the manufacturer’s protocol for seven independent *LOG* OX lines, and this experiment was repeated three times.

### Chlorophyll Extraction and Quantification

Chlorophyll was extracted from *LOG* OX and WT cells using 80% methanol. The total chlorophyll content (a + b) was calculated in five independent LOG OX lines (three biological replicates each) by absorbance taken at 666, 653, and 470 according to equations described in an earlier study (Lichtenthaler and Wellburn, 1983).

### Cell Cycle Analysis

Wild type and *LOG* OX1 cells were collected at two time points: 5 h after the dark phase and 1 h before the dark phase, each with three biological replicates, and this experiment was repeated three times. These cells were pelleted down at 5,000 × *g* for 5 min, and the supernatant was discarded. The pellet was washed with 1 ml of 1 × PBS (pH 7.6), and finally, the pellet was resuspended in 1 ml of 70% ethanol and stored at 4°C until further use. For staining, the 70% ethanol was removed. The pellet was washed with 1 × TBS (pH 7.6) and stained with 1 μl of working concentration of DAPI (Sigma, United States) in 1 ml of 1 × TBS for 30 min at room temperature, which was followed by flow cytometric analysis in the Aria II Flow cytometer (BD Biosciences) as per manufacturer’s protocol.

### Gravimetric Dry Weight Estimation

Biomass production in milligrams dry weight (mg dw)/L of culture was estimated for flask cultures by collecting 10 ml of stationary phase cultures (19 dpi; WT, 29 dpi; *LOG* OX1) for three biological replicates each by a method described in an earlier study (Chioccioli et al., 2014). The culture was centrifuged (1,500 × *g*, 5 min) in sterile 15-ml tubes (Tarsons). The pellet was then gently washed with 2 ml of sterilized deionized water to remove the culture medium. The sample was pelleted again (1,500 × *g*, 5 min). The supernatant was carefully removed, and 1.5 ml of sterilized deionized water was used in several aliquots to re-suspend the pellet into pre-weighed 1.5-ml Eppendorf tubes. Cells were pelleted at 1,500 × *g* for 5 min, and the supernatant was discarded. The pellets were dried in a hot air oven (ThermoScientific, United States) at 60°C. Tubes were weighed on a precision balance (Shimadzu, Japan) to estimate the weight of the dried biomass. This experiment was repeated three times.

### Sample Preparation for Cell Composition Analysis

Sample preparation was done according to a previously described method from WT and LOG OX1 cultures (Li et al., 2015). At the end of the cultivation, 1 ml of sample was centrifuged at 15,000 × *g* for 10 min in a 2-ml tube, the supernatant was discarded, and 1 ml of deionized water was added to wash the pellet using vortex for 5–10 s. The sample was then centrifuged at 15,000 × *g* for 10 min, and the supernatant was discarded. The pellet was then snap frozen in liquid nitrogen. The 2-ml tube containing the sample was then stored in darkness at −20°C until analysis.

### Total Carbohydrate Analysis

Total carbohydrate analysis was done for WT and *LOG* OX1 stationary phase cultures (19 dpi; WT, 29 dpi; *LOG* OX1) with three biological replicates each according to a method described in an earlier study (Li et al., 2015). One molar H_2_SO_4_ (1.8 ml) was added to the sample, mixed well by vortexing, heated in a water bath at 95°C for 2 h, cooled to room temperature, and centrifuged at 15,000 × *g* for 10 min. The supernatant was used for carbohydrate measurement by phenol-sulfuric acid method. Of the supernatant or standard glucose solution, 0.4 ml was added to 0.2 ml of 5% phenol solution in a 2-ml tube. Subsequently, 1 ml of concentrated sulfuric acid was added quickly and mixed well by vortexing instantly. After cooling the tube for 30 min at room temperature for color development, 200 μl of the solution was pipetted into the bottom of a 96-well microplate (Corning, United States), and absorbance was measured at 490 nm by Infinite M200 pro plate reader (Tecan, Switzerland). This experiment was repeated three times.

### Total Lipid Analysis

The lipid content was measured by the colorimetric sulfo-phospho-vanillin (SPV) method for WT and *LOG* OX1 stationary phase cultures (19 dpi; WT, 29 dpi; *LOG* OX1) with three biological replicates each according to a method described in an earlier study (Mishra et al., 2014) with some modifications (Li et al., 2015). The standard lipid stocks were made by dissolving 10 mg of corn oil (Sigma–Aldrich) in 10 ml of chloroform. Different amounts of standard stocks containing 50–500 μg lipids were added to the bottom of a 2-ml tube. The solvent was dried in an incubator (ThermoScientific, United States) at 30°C. Subsequently, 100 μl of deionized water was added to the 2-ml tube containing a known amount of algal biomass or corn oil. Concentrated sulfuric acid (1.4 ml) was added and mixed well by vortexing; then the mixture was heated for 10 min at 100°C, cooled in an ice bath for 5 min, and mixed again. Of the mixture, 1.8 ml was pipetted into the bottom of microplate wells. Background absorbance at 530 nm was measured. Vanillin-phosphoric acid reagent (1.2 mg vanillin per ml of 68% phosphoric acid) (100 μl) was added to each well for color development for 20 min, and the absorbance was measured at 530 nm by Infinite M200 pro plate reader (Tecan, Switzerland). This experiment was repeated three times.

### Cell Counting Using Flow Cytometry

Absolute microalgae cell counts were obtained for WT and *LOG* OX1 cultures at 14 dpi and stationary phase for three biological replicates each by a method described in an earlier study (Chioccioli et al., 2014). Absolute microalgae cell counts were obtained by adding an internal microsphere cell counting standard (Count Bright; Invitrogen, CA) to the flow cytometric sample (single platform testing). A 1:9 dilution of Count Bright suspension was added to the microalgae culture. Using a FSC-A vs. SSC-A plot to separately gate cell events vs. bead events, the ratio of bead events to cell events (together with the known concentration of beads) was used to calculate the absolute cell concentration. This experiment was repeated three times.

### RNA Isolation

Total RNA was isolated from the 14 dpi wild type and five independent *LOG* OX lines (three biological replicates each) using Trizol reagent (Invitrogen, United States), following the manufacturer’s instructions (Invitrogen) as described earlier (Chomczynski and Sacchi, 1987). The quality of RNA samples was assessed using agarose gel electrophoresis. The RNA samples with O.D. ratios at 260/280 nm in the range of 1.9–2.1 and 260/230 nm in the range of 2.0–2.3 (ND-1000 Spectrophotometer, ThermoScientific, United States) were used for cDNA preparation. For RNA sequencing, RNA was isolated from three biological replicates of WT and LOG OX1.

### Quantitative PCR

The cDNA of wild type and *LOG* OX for qPCR was synthesized using total RNA using the cDNA archive kit (ThermoFisher Scientific, United States) as per manufacturer’s protocol from five independent lines, with three biological replicates each for confirming CvarLOG1 overexpression. For validation of RNA-Seq results by qPCR, cDNA was synthesized from WT and *LOG* OX1 from three biological replicates each. Real-time qPCR primer designing was done by using Primer 3 plus software, and the primers (see Supplementary Table 5) were confirmed to be unique using the BLASTn tool, NCBI. qPCR reactions were carried out according to the manufacturer’s protocol for the three biological replicates along with three technical replicates each (SYBR green, ThermoFisher Scientific, United States). The relative abundance of the transcript was calculated as per the method described previously, and 18 s rRNA was used as the internal control (Livak and Schmittgen, 2001; Derveaux et al., 2010).

### RNA Sequencing

Total RNA was extracted using Trizol (Invitrogen, United States) from *Chlorella* cells (WT and *LOG* OX1, three biological replicates each) as per the manufacturer’s protocol. RNA-sequencing libraries were prepared with Illumina-compatible NEB Next^®^ Ultra^TM^ II Directional RNA Library Prep Kit (New England Bio Labs, United States) at Genotypic Technology Pvt. Ltd., Bangalore, India, following manufacturer’s instructions. The Illumina-compatible sequencing library was quantified by Qubit fluorometer (ThermoFisher Scientific, United States), and its fragment size distribution was analyzed on Agilent 2200 tape station. The average fragment size across the libraries was observed to be 500 bp, with an average Qubit-based concentration of 11.6 ± 3 ng/μl. The samples were molar normalized and pooled for multiplexed paired-end sequencing on Illumina HiSeq X Ten sequencer. A total of 35.6 Gbp were generated across the six samples sequenced, with a mean quality score > 37, and >88% bases called with Phred score *Q* > 30. The raw data generated was checked for the quality using FastQC2^2^. Reads were pre-processed to remove the adapter sequences and removal of the low-quality bases (<q30). Pre-processing of the data is done with Trimgalore3.^3^ HISAT-24, which is a splice aligner (Kim et al., 2015), was used to align the high-quality reads to the reference *Chlorella variabilis* NC64A genome^4^ downloaded from NCBI database with the default parameters. HTSeq was used to estimate and calculate gene abundance (Anders et al., 2015). Absolute counts for each gene were identified, which were used in differential expression calculations. DESeq was used to calculate the differentially expressed genes (DEGs) (Anders and Huber, 2010). Genes were categorized into up- and downregulated based on the fold change cut off of 1.5, adjusted *p*-value (*q*-values) ≤ 0.05. For each gene, gene ontology (GO) was assigned based on the homology search against algae reviewed protein sequences downloaded from the Uniprot database. Reference sequences were matched against Uniprot data using the Diamond BLAST program (Altschul et al., 1990). These GO terms were mapped to the differentially expressed (DE) genes. Pathways for each gene were obtained from the KEGG KAAS 5 server (Moriya et al., 2007). Compiled pathways per gene were mapped to the DEGs. The RNA-Seq, raw and processed files have been deposited to GEO database, NCBI with accession number GSE162985.

### Statistics

Each experiment had three biological replicates for each sample, and the statistical significance of the results was calculated using the Student’s two-tailed, unpaired *t*-test where *p*-value < 0.05 was considered to be significant.

## Results and Discussion

### *Chlorella variabilis* NC64A Has a Single LONELY GUY Ortholog

**FIGURE 1:**
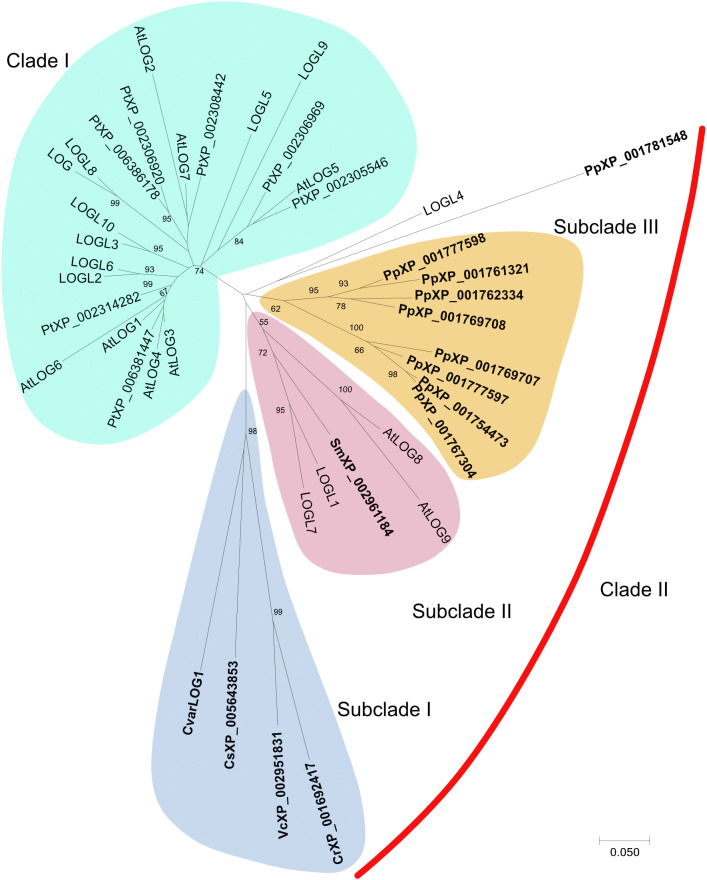
Unrooted phylogenetic tree of CvarLOG1 and homologs in seed plants *Arabidopsis* (At), rice (Os), *Populus trichocarpa* (Pt), and non-seed plants such as *Selaginella moellendorffii* (Sm), *Physcomitrella patens* (Pp), *Coccomyxa subellipsoidea* (Cs), *Volvox carteri* (Vc), and *Chlamydomonas reinhardtii* (Cr). Full-length protein sequences of all the LOG homologs were obtained from protein databases. The phylogenetic tree was constructed in the MEGA 7.0 program (http://www.megasoftware.net) using the neighbor-joining method. The phylogenetic tree was built with 1,000 bootstrapping replicates. The bootstrap values of 50% and above are indicated on the tree. The scale bar represents 0.05 amino acid substitutions per site. The non-seed LOG proteins are highlighted in bold.

*CvarLOG1* codes for a putative cytokinin riboside 5′-monophosphate phosphoribohydrolase, a cytokinin-activating enzyme and commonly known as LONELY GUY (LOG). *CvarLOG1* was identified using BLASTp analysis (NCBI) with AtLOG3 as the query sequence. There is only one such gene in *Chlorella variabilis*, and it shares 54% identity to the AtLOG3 protein with a query coverage of 87%. The 211-amino acid sequence of this protein was aligned with other LOG proteins from different seed plants such as *Populus trichocarpa*, Rice, *Arabidopsis*, and non-seed plants such as *Selaginella moellendorffii*, *Physcomitrella patens*, *Coccomyxa subellipsoidea*, *Volvox carteri*, and *Chlamydomonas reinhardtii* (Supplementary Table 1). Phylogenetic analysis of the selected LOG proteins from both seed and non-seed plants showed that they formed two major clades. Clade I exclusively consists of LOGs from seed plants, whereas CvarLOG1 protein forms a part of subclade I in Clade II along with other algal proteins (Figure 1). It is interesting to note that in clade II, there are three major subclades where subclades I and III exclusively belong to non-seed plants, and subclade II has *Selaginella*, *Arabidopsis*, and Rice proteins. Thus, in clade II, LOG proteins of non-seed plants and seed plants maybe functionally similar because of their structural similarities. All the selected LOG proteins show highly conserved regions (Supplementary Figure 1). A conserved domain search on NCBI revealed the presence of a lysine decarboxylase (LDC) domain in CvarLOG1 with a conserved PGGxGTxxE motif like the LOG counterparts from seed plants (Supplementary Figure 2). To date, PGGxGTxxE motif-containing LDCs do not show LDC activity; they instead show phosphoribohydydrolase activity (Naseem et al., 2018). Hence CvarLOG1 is structurally comparable to the known LONELY GUY enzymes. In *Chlorella variabilis* NC64A, there is only one LONELY GUY-like protein, whereas this cytokinin-related gene has evolved into a gene family in *Physcomitrella patens* and seed plants (Kurakawa et al., 2007; Kuroha et al., 2009; Chickarmane et al., 2012; Tokunaga et al., 2012; Lu et al., 2014).

### CvarLOG1 Is a Cytokinin-Activating Enzyme

The CvarLOG1 protein was tagged with His, expressed, and purified from *E. coli* to study the involvement of CvarLOG1 in catalyzing inactive cytokinin nucleotides to active free-base cytokinins (Supplementary Figure 3). The recombinant protein was used to check its enzymatic activity in the presence of the substrate; Iprmp, and tZRMP. A control reaction without enzyme was included to confirm the substrate conversion. The products were detected by UHPLC and compared to the standards.

**FIGURE 2:**
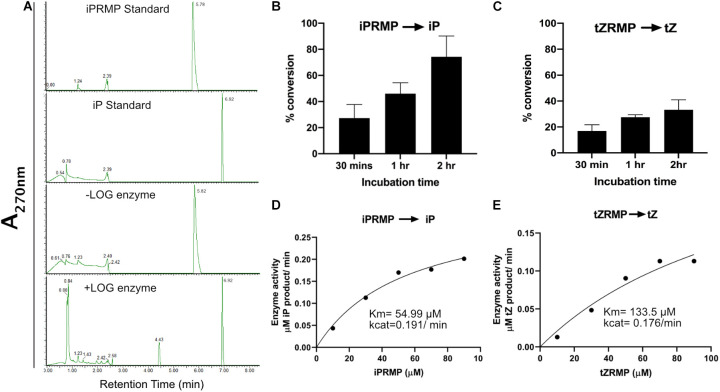
Enzyme assay of CvarLOG1; detection and identification of reaction products. **(A)** Standards iPRMP and iP, reaction products of iPRMP without LOG and with LOG were separated by UHPLC. Phosphoribohydrolase activity of CvarLOG1 using the substrates **(B)** iPRMP and **(C)** *t*ZRMP as measured by UHPLC in terms of percent conversion; bars indicate ± SE (*n* = 2). For control, a without enzyme sample was used. Determination of CvarLOG1 kinetic parameters for the conversion of **(D)** iPRMP to iP and **(E)** *t*ZRMP to tZ.

From these results, it was evident that CvarLOG1 can catalyze the enzymatic reaction where cytokinin nucleotides such as iPRMP and *t*ZRMP are converted to the free-base form iP and *t*Z, respectively (Figures 2A–C). The rate of conversion for the *t*ZRMP substrate was much lower than the iPRMP substrate (Figure 2C). CvarLOG1 had a Km value of 54.99 μM for iPRMP (Figure 2D), and the Km value for *t*ZRMP (133.5 μM) was much higher, suggesting that the CvarLOG1 enzyme affinity for *t*ZRMP is much lower (Figure 2E). CvarLOG1 recombinant protein with iPRMP as the substrate showed almost four times more Km value than the Rice LOG, AtLOG3, and AtLOG5, and had a comparable Km value with AtLOG2 (Kurakawa et al., 2007; Kuroha et al., 2009). The predominant cytokinin in microalgae is *cis*-zeatin followed by iP, with low levels of *t*Z (Stirk et al., 2013b), and thus, CvarLOG1 enzyme probably has a better affinity for iPRMP than *t*ZRMP. Hence, like the LOG enzymes of rice and *Arabidopsis*, the LOG enzyme of *Chlorella variabilis* NC64A is also involved in cytokinin activation. Thus, the LOG enzymes are functionally conserved from unicellular algae to higher plants.

### Expression Analysis of *CvarLOG1*

**FIGURE 3:**
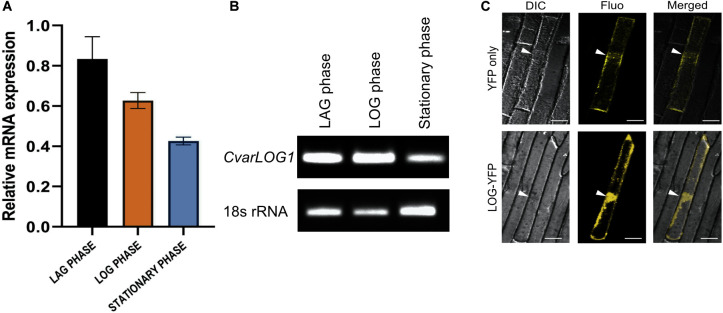
Expression analysis of *CvarLOG1* and *in vivo* localization CvarLOG1 protein. The levels of *CvarLOG1* mRNA were checked across the growth phases of the alga. The growth phases can be divided into lag, log, and stationary phases. The levels of *CvarLOG1* were quantified using total RNA extracted from each of these phases by **(A)** Quantitative PCR; bar indicates ± SE (*n* = 3). **(B)** Semi-quantitative PCR, for both qPCR and semi-qPCR, 18s rRNA was used as the internal standard. **(C)** Sub-cellular localization of CvarLOG1-YFP fusion protein and YFP protein was done in onion epidermal cells transiently by particle bombardment and visualized using confocal microscopy. White arrows showing the nucleus. DIC, differential interference contrast; Fluo, fluorescence; Merged, overlay of DIC and Fluo. Bar = 50 μm.

In this study, the growth of *Chlorella variabilis* NC64A has been divided into three phases: lag phase, log phase, and stationary phase (Supplementary Figure 4). The *CvarLOG1* expression was studied using qPCR and semi-quantitative PCR during these three stages. *CvarLOG1* has higher expression during the lag and log phase, whereas its expression diminishes during the stationary phase (Figures 3A,B). These results suggest that *CvarLOG1* may have a role during the active cell division and cell growth phase. In rice, the expression of *LOG* was detected in the majority of meristem regions, and its signal was localized strongly in two or three layers of cells at the tip of the meristem, hence suggesting a role for it in meristem maintenance (Kurakawa et al., 2007). In *Arabidopsis*, the septuple mutants of the LOG family revealed a drastic reduction in apical meristems, thus demonstrating its active role during meristem maintenance (Tokunaga et al., 2012). In *Medicago truncatula*, two *LOG* genes were shown to be induced during nodulation in a CRE1-dependent manner, and their expression was primarily limited in the dividing cells of the nodule primordium (Mortier et al., 2014). Taken together, *CvarLOG1* expresses during the active cell division and growth phase, and *LOG* genes of seed plants express mainly in the meristem, a tissue responsible for cell division and cell growth. Hence, the expression domain of LOG genes seems to have remained conserved from unicellular microalgae to the seed plants.

To check the *in vivo* localization of CvarLOG1, YFP was fused to the protein at its C terminal end and expressed transiently in onion epidermal cells. The CvarLOG1 protein was seen to localize in both nucleus and cytosol (Figure 3C). Transient expression studies of the *Arabidopsis* LOG-GFP fusion proteins in *Arabidopsis* root cells revealed that these proteins localize in both nucleus and cytosol (Kuroha et al., 2009). However, the rice LOG-GFP fusion protein localized to the cytosol as determined by transient assays in onion epidermal peel cells (Kurakawa et al., 2007). Hence, like the counterpart proteins of *Arabidopsis*, CvarLOG1 also displays a similar pattern of localization in the onion epidermal cells (Kuroha et al., 2009).

### Overexpression of *CvarLOG1* (*CvarLOG1 OX*)

**FIGURE 4:**
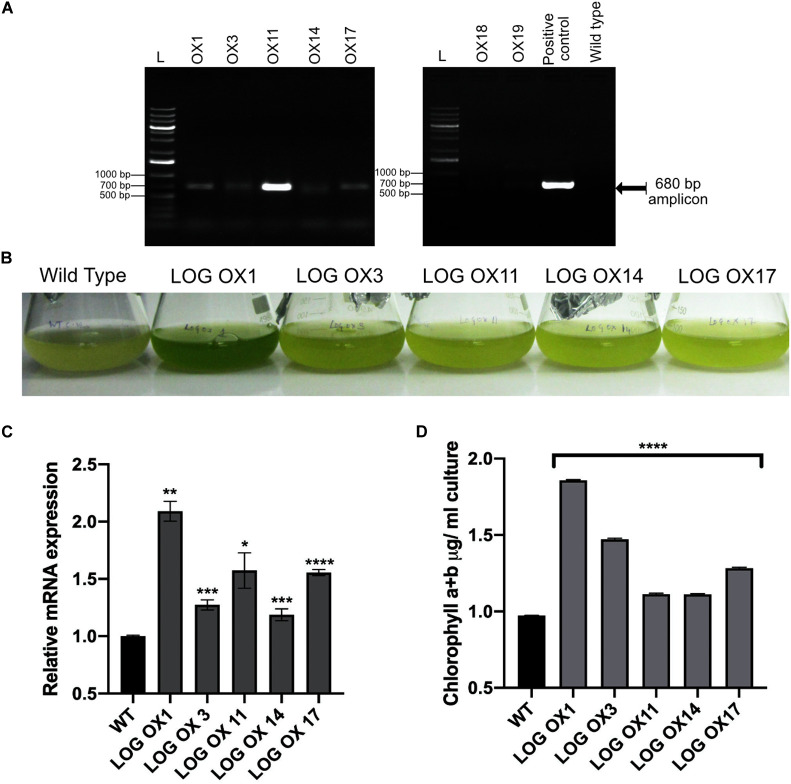
Overexpression phenotype of the *CvarLOG1.* **(A)** Colony PCR confirmation of seven independent transgenic lines versus the wild type was compared to the positive control. An amplicon of size 680 bp was seen in all lines and positive control, faints bands were detected in lines OX18 and OX19. **(B)** *LOG* OX phenotype 30 dpi (days post inoculation) where all five independent OX lines have a stay-green phenotype and wild type has started senescing; *LOG* OX1 showed the best phenotype. **(C)** *CvarLOG1* transcript accumulation in wild type and five independent OX lines during log phase as determined by qPCR; bars indicate ± SE (*n* = 3). **(D)** Chlorophyll content of WT and five independent *LOG* OX cultures at 30 dpi; bars indicate ± SE (*n* = 3). For all graphs, data were significant at ****P ≤ 0.0001, ***P ≤ 0.001, **P ≤ 0.01, and *P ≤ 0.05 compared with the wild type using Student’s *t*-test.

Previous studies by exogenous application of cytokinin in microalgae have revealed that growth rate, oil content, cell cycle progression, and stress tolerance were enhanced (Stirk et al., 2011, 2014; Park et al., 2013; Lu et al., 2014). Since *CvarLOG1* is a cytokinin-activating gene, it was checked by overexpression (OX) if it led to any changes in the phenotype in terms of the traits mentioned above. *CvarLOG1* was cloned in binary vector pMDC32 vector with a constitutive 35S promoter and was transformed in wild type *Chlorella* cells by electroporation. There were 22 stable transgenics after eight rounds of selection, out of which seven independent lines were then grown in liquid media for further studies. The integration of the gene containing cassette was confirmed by colony PCR with hygromycin primers in the seven independent lines (Figure 4A), and the overexpression in five of these lines was confirmed by qPCR (Figure 4C). The observed CvarLOG1 overexpression was not very high; in *LOG* OX1, *LOG* OX11, and *LOG* OX17, it was 1.5- to 2-fold more than the wild type, but with a very evident phenotype. In cultures 30 days post-inoculation (dpi), it can be observed that the wild type culture had started senescing.

In contrast, the *CvarLOG1 OX (LOG OX)* lines were still green, as visible from the difference in intensity of chlorophyll retention (Figure 4B). The *LOG OX* lines had 14–90% more chlorophyll compared to the WT at 30 dpi (Figure 4D). The LOG OX lines have been maintained for 64 generations without any loss of phenotype and are continuing to show the same phenotype. All the LOG OX lines survived 10–14 days more than the WT, but the line *LOG* OX1 survived for almost 14–20 days more than the WT. Hence, LOG OX1 was used for all further analysis as it displayed the best phenotype (Supplementary Figure 5). Plants with higher levels of cytokinin show a “stay-green phenotype” (Thomas and Howarth, 2000), which was visible in all the *LOG* OX lines. The presence of this phenotype raises the chance of *LOG* OX lines having higher levels of cytokinin compared to the wild type, as observed for the LOG overexpression plants of *Arabidopsis* (Kuroha et al., 2009).

### LOG OX Has an Extended Life in the Culture

**FIGURE 5:**
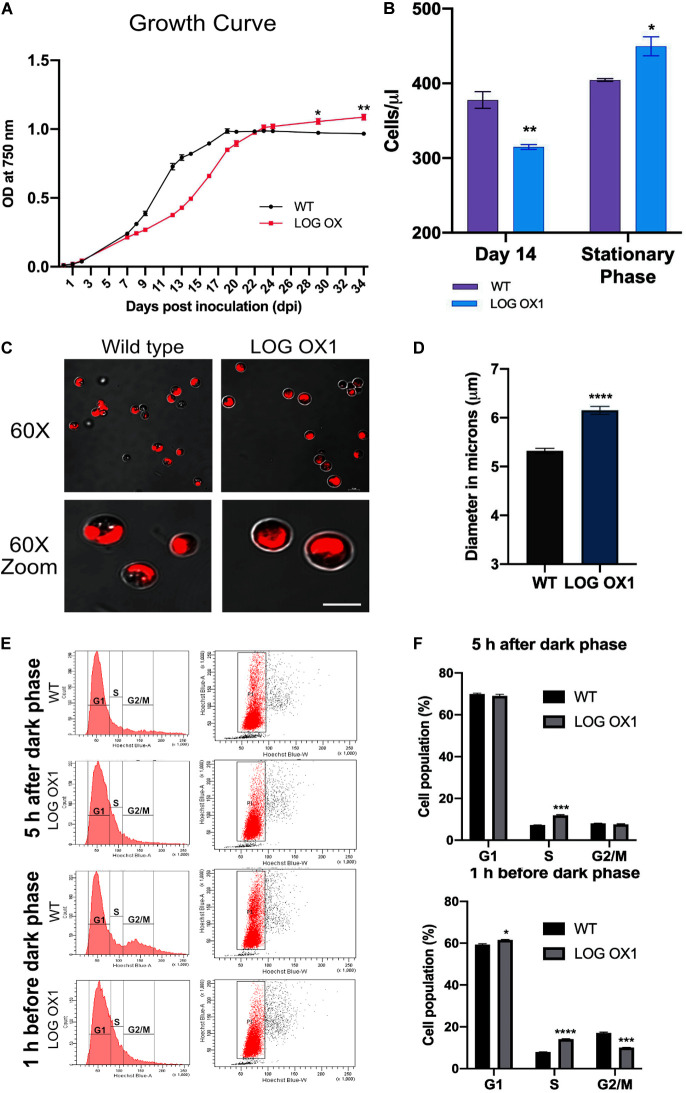
Detailed characterization of the *LOG* OX1 phenotype. **(A)** Spectrophotometric values of optical density (OD) at 750 nm was plotted versus time (dpi; days post inoculation) to determine the growth kinetics of *LOG* OX vs. WT, bars indicate ± SE (*n* = 3). **(B)** Absolute cell counts of *LOG* OX1 and WT algae to determine cell number at 14 dpi and stationary phase (19 dpi; WT, 29 dpi; *LOG* OX1, bars indicate ± SE, *n* = 3). **(C)** Comparison of cells of wild type and *LOG* OX1 morphology at 20 dpi, visually the cells of *LOG* OX are more prominent in size. Bar = 5 μm **(D)** Comparison of cell diameter in wild type and *LOG* OX1 cells at 20 dpi, bars indicate ± SE (*n* = 3, with at least 50 cells in each replicate). **(E)** Cell cycle analysis for 14 dpi culture from 5 h after dark phase and 1 h before dark phase of wild type and *LOG* OX1 cells. Cells were stained with DAPI and monitored using a flow cytometer. **(F)** Cell cycle phase distribution in terms of percentage in WT and *LOG* OX1 for samples taken from 5 h after dark phase and 1 h before dark phase 14 dpi cultures; bars indicate ± SE (*n* = 3). For all graphs, data significant at ****P ≤ 0.0001, ***P ≤ 0.001, **P ≤ 0.01, and *P ≤ 0.05 compared with the wild type using Student’s *t*-test.

The growth kinetics of the WT and *LOG* OX1 cells was monitored from 1 to 34 dpi by measuring the optical density (OD) at 750 nm as an increase in OD relates to increasing in biomass for a culture that shares average cell characteristics (Griffiths et al., 2011; Chioccioli et al., 2014). For all the remaining experiments, three biological replicates each of WT and *LOG* OX1 were taken, and these experiments were repeated three times to test the repeatability. The stationary phase of the wild type was observed at 19 days post-inoculation (dpi), and 28–29 dpi for *LOG* OX1. The growth of *LOG* OX1 cells was slower than the wild type, and OD_750_ values of *LOG* OX1 for the period 7–20 dpi were lesser than the WT. Later at 23–34 dpi, the OD_750_ values of *LOG* OX1 cultures were now more than WT as the growth of the *LOG* OX1 cells slowly gained momentum whereas in WT cultures, growth declined (Figure 5A). Hence, due to LOG overexpression, these lines remained green and exhibited an extended log phase, which may have led to a longer stationary phase as senescence was impeded. In contrast, the counterpart wild type had already started senescing and turning pale yellowish green by 23–29 dpi; *LOG* OX1 remained green till 37–50 dpi, thus pointing toward the probability that the stationary phase was longer in these cultures (Supplementary Figure 5). The work done in this study is focused only till 29–30 dpi, though further increase in OD_750_ was observed even at 34 dpi (Figure 5A).

The absolute microalgae cell counts (Chioccioli et al., 2014) were also measured to validate the changes in growth patterns among WT and *LOG* OX1. This was done by adding an internal microsphere cell counting standard to the diluted WT and *LOG* OX1 cultures and collecting data for 1,000 bead events in the flow cytometer. During the slow growth phase of *LOG* OX1 (14 dpi), its cell number was lesser than the wild type (Figure 5B). The cell number in *LOG* OX1 cultures was 11% greater than the WT during their respective stationary phases (Figure 5B), which was also evidenced by the increase in OD_750_ values (Figure 5A). It was interesting to note that the size of the cells in *LOG* OX1 had also increased by almost 15–20% at 20 dpi (Figures 5C,D). Since there was slower growth of *LOG* OX1 for the period of 7–20 dpi, the status of the cell cycle was studied in 14 dpi cultures of WT and *LOG* OX1 at 5 h after the dark phase and 1 h before the dark phase. At 5 h after the dark phase, it was observed that majority of WT and *LOG* OX1 cells in the gated sub-population P1 were in G1 phase, whereas the S phase (7.2%) of WT was lesser than *LOG* OX1 (11.9%) with an equal percentage of both WT and *LOG* OX1 cells during G2/M phase (Figures 5E,F). At 1 h before the dark phase in wild type, the cells in the gated sub-population P1 were seen shifting toward the G2/M phase, whereas in *LOG* OX1, an increase in the percentage of cells in the S phase was observed (Figures 5E,F). The following are the percentage distribution of phases in *LOG* OX1 (gated sub-population P1): G1 phase (61.5%), S phase (14.1%), and G2/M phase (10%), and in WT: G1 phase (59.3%), S phase (7.9%), and G2/M phase (17%) at 1 h before dark phase (Figures 5E,F). This showed that overexpression of *LOG* had a positive effect on the G1/S phase transition during the light cycle, although there was a reduction in the G2/M phase toward the end of the light cycle. According to several studies, cytokinin plays a decisive role in G1/S and G2/M progression (Laureys et al., 1998; Riou-Khamlichi et al., 1999; Francis, 2011; Lipavska et al., 2011). In *LOG* OX1, further progression to G2/M may have been delayed due to cytokinins exceeding the optimal levels required for this transition (Schaller et al., 2014), which explains the slower growth of *LOG* OX1 during 7–20 dpi. In BY2 tobacco cell lines, it is known that cytokinin alternations are responsible for the progression of the cell cycle; hence, any drastic change in its levels may cause a block or delay of the cell cycle (Laureys et al., 1999). Thus, constitutive expression of *LOG* may be producing levels of cytokinin unsuitable for cell cycle progression and causing a delay in cell division. The delayed progression to G2/M in *LOG* OX1 leads to the extended life of the alga in culture. There is also increased cell number during the stationary phase due to continued growth and delayed senescence of these cultures, which is a direct effect of overexpression of *CvarLOG1*, whereas in WT, growth declined. Thus, this evidence supports the positive influence of CvarLOG1 enzyme on the longevity of the culture, which ultimately leads to increased cell numbers in the stationary phase, as observed for LOG overexpression plants of *Arabidopsis* (Kuroha et al., 2009).

### LOG OX1 Accumulates More Biomass, Total Carbohydrates, and Lipids Than Wild Type During Its Stationary Phase

**FIGURE 6:**
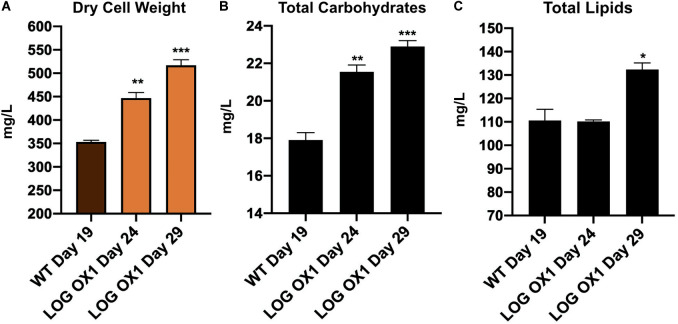
Biomass and biochemical composition of *LOG* OX1 and WT cultures. **(A)** Biomass in terms of dry cell weight at the stationary phase of WT (19 dpi; days post inoculation) and *LOG* OX1 (29 dpi) cultures; bars indicate ± SE (*n* = 3). **(B)** Total carbohydrates in terms of carbohydrate yield at the stationary phase of WT (19 dpi) and *LOG* OX1 (29 dpi) cultures; bars indicate ± SE (*n* = 3). **(C)** Total lipids in terms of lipid yield at the stationary phase of WT (19 dpi) and *LOG* OX1 (29 dpi) cultures; bars indicate ± SE (*n* = 3). For all graphs, data significant at ***P ≤ 0.001, **P ≤ 0.01, and *P ≤ 0.05 compared with the wild type using Student’s *t*-test.

Since the OD_750_ values and cell numbers were more in the case of *LOG* OX1 compared to the wild type during their respective stationary phases, the biomass of *LOG* OX1 versus WT in terms of dry cell weight was measured. *LOG* OX1 had an almost 46% increase in biomass compared to the wild type strain during their respective stationary phases without any extra effort, such as changes in medium composition or any external input (Figure 6A). The increase was also evident on day 24 in *LOG* OX1 (Figure 6A).

An increase in biomass indicates that there should be an associated increase in the level of macromolecules. The level of total carbohydrates was checked using the phenol-sulfuric acid method in wild type (19 dpi) and *LOG* OX1 (29 dpi). There was approximately 30% increase in carbohydrate yield in *LOG* OX1 compared to the WT (Figure 6B). The level of total lipids was checked using the colorimetric sulfo-phospho-vanillin (SPV) method in wild type (19 dpi) and *LOG* OX1 (29 dpi). There was an approximately 20% increase in lipid yield in *LOG* OX1 compared to the WT (Figure 6C). The increase in carbohydrate and lipid yield was not comparable to the increase in biomass, which is often observed in high biomass-producing strains as both biomass and lipid pathways compete for the same substrate (Tan and Lee, 2016). Therefore, for application in biofuel, instead of downregulating starch synthesis that may be disadvantageous for growth, it may be better to direct the flow of photosynthetic carbon to lipid biosynthesis rather than starch in these high biomass-producing strains (Griffiths and Harrison, 2009; Hempel et al., 2012; Tan and Lee, 2016). Another reason for this may be due to prolonged incubation in the same medium that may have affected essential culture conditions like CO_2_ levels, pH, and nutrient availability (Alishah Aratboni et al., 2019). These factors, which were not altered in this study, play a significant role in increasing lipid levels (Alishah Aratboni et al., 2019). In the future, a permutation of the conditions mentioned above, along with the impact of nutrient limitation, can be studied to enhance the lipid levels of *LOG* OX1 for use in developing biofuel.

### Pathways Affected by LOG Overexpression

**FIGURE 7:**
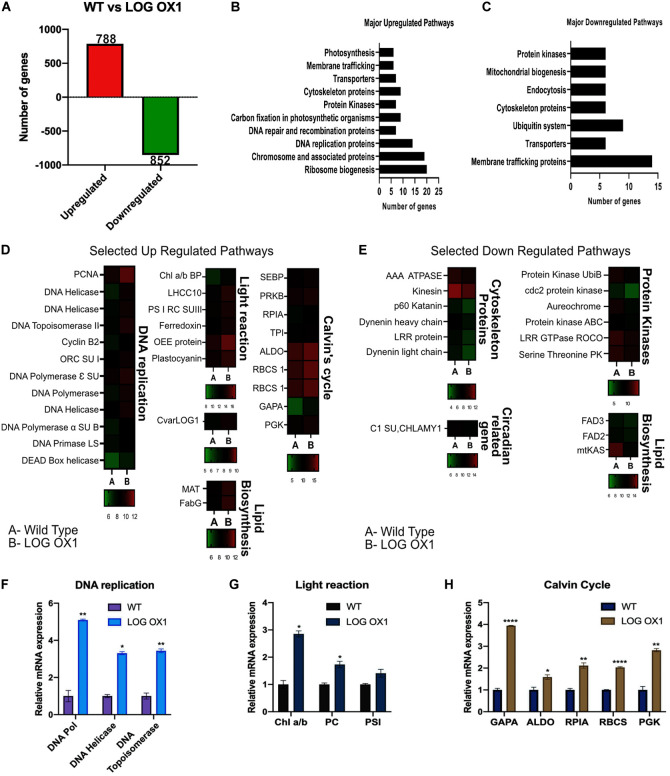
RNA-Seq based comparison of *LOG* OX1 and WT transcriptome at 14 days post inoculation. **(A)** Total number of differentially expressed genes (1.5 fold) in *LOG* OX in contrast to the wild type; upregulated genes (red) and downregulated genes (green) (FDR ≤ 0.05, *n* = 3). **(B)** Major upregulated pathways in *LOG* OX1 in comparison to WT. **(C)** Major downregulated pathways in *LOG* OX1 in comparison with WT. **(D)** Heat maps of selected upregulated pathways in *LOG* OX1; color bars below each sub-panel represent the range of expression values in log_2_. **(E)** Heat maps of selected downregulated pathways in *LOG* OX1; color bars below each sub-panel represent the range of expression values in log_2_. Real-time quantitative PCR validation of RNA Seq data for selected genes from **(F)** DNA replication. **(G)** Light reaction of photosynthesis. **(H)** Dark reaction of photosynthesis; bars indicate ± SE (*n* = 3). For all graphs, data significant at ****P ≤ 0.0001, **P ≤ 0.01, and *P ≤ 0.05 compared with the wild type using Student’s *t*-test.

To assess the significant downstream pathways affected by the overexpression of *LOG* in *Chlorella*, transcriptome analysis was carried out using RNA sequencing of three biological replicates each from WT and *LOG* OX1 at 14 dpi (1 h before dark phase). The 14 dpi was chosen for the analysis because it was in the middle of the growth phase, where the slower growth of *LOG* OX1 was evident. According to RNA Seq data, CvarLOG1 is upregulated in LOG OX1 versus WT by 1.8-fold, thus correlating with the qPCR data (Figure 7D and Supplementary Table 3). The RNA sequencing results of *LOG* OX1 versus WT at 14 dpi confirmed that a total of 1,640 genes were differentially expressed [1.5-fold change at adjusted *p*-value (*q*-values) ≤ 0.05]: 788 genes were upregulated, and 852 were downregulated (Figure 7A and Supplementary Table 2). From these DEGs, 176 upregulated genes and 174 downregulated genes had algae reviewed protein functions in the UniProt Database. Further, from this subset, only 128 upregulated and 102 downregulated genes could be mapped to pathways in the KEGG database. The major pathways upregulated in the *LOG* OX1 include chromosome and associated proteins (19 genes), DNA replication (14), transporters (7), photosynthesis; light reaction (6) and dark reaction (9), ribosome biogenesis (20), and related genes (Figure 7B and Supplementary Table 3, certain genes belong to more than one pathway). The highest number of upregulated genes (20) belonged to the ribosome biogenesis pathway. Cytokinin has been shown to induce genes involved in ribosome biogenesis (Cherepneva et al., 2003; Brenner et al., 2005; Brenner and Schmulling, 2012). Thus, in *LOG* OX1, there may be heightened levels of cytokinin that could be inducing the ribosome biogenesis pathway.

DNA replication genes such as *DNA POLYMERASE, DNA HELICASE*, and *DNA TOPOISOMERASE* were upregulated along with 11 other genes from this pathway (Figures 7D,F). The differential expression of three genes from this pathway was also confirmed by qPCR (Figure 7F and Supplementary Table 3). DNA replication genes are expressed maximally in the early S phase (van der Meijden et al., 2002), and in *LOG* OX1, the percentage of cells in the S phase was higher than in the wild type (Figures 5E,F). This result thus correlates with the upregulation of the genes from the DNA replication pathway.

From the fatty acid biosynthesis pathway, two genes were upregulated; *MAT* gene (*MALONYL-COA: ACP_TRANSACYLASE*) and *ß-KETOACYL (ACP) REDUCTASE* (Figure 7D). The *MAT* gene is responsible for the synthesis of malonyl-ACP, involved in the initiation step for fatty acid biosynthesis (Li et al., 2018). The PUFA levels of *Schizochytrium* sp. increased when this gene was overexpressed (Li et al., 2018). The effect of upregulation of MAT gene remains to be studied in *LOG* OX1.

The upregulation of six light reaction-related genes was observed in *LOG* OX1 (Figures 7D,G and Supplementary Table 3). The upregulation of *CHLOROPHYLL A/B BINDING PROTEIN*, *PHOTOSYSTEM I REACTION CENTER SUBUNIT III*, and *PLASTOCYANIN* (PC) was also confirmed by qPCR (Figure 7G). Cytokinin has been shown to regulate chloroplast development and function positively (reviewed in Cortleven and Schmulling, 2015). It has been shown earlier that cytokinin can induce some of the light reaction genes (Brenner and Schmulling, 2012). In *Arabidopsis*, there are two genes encoding for plastocyanin, *PETE1* and *PETE2*. The overexpression of both genes leads to an unexplained increase in size and dry weight of leaves, indicating an increase in biomass (Pesaresi et al., 2009). *PLASTOCYANIN* upregulation in *LOG* OX1 may thus have a similar consequence.

The majority of Calvin’s cycle genes were upregulated in *LOG* OX1 (Figures 7D,H and Supplementary Table 3). Upregulation of *RIBULOSE-BISPHOSPHATE CARBOXYLASE SMALL CHAIN (RBCS)*, *PHOSPHOGLYCERATE KINASE (PGK)*, *GLYCERALDEHYDE-3-PHOSPHATE DEHYDROGENASE (GAPA)*, *RIBOSE 5-PHOSPHATE ISOMERASE A (RPIA)*, and *FRUCTOSE-BISPHOSPHATE ALDOLASE (ALDO)* was also confirmed by qPCR (Figure 7H). Some of the Calvin cycle genes are positively regulated by cytokinin application, as observed by previous studies (Brenner and Schmulling, 2012). Calvin cycle genes like *GAPA* (also a carbon partitioning enzyme), *RBCS*, and *ALDO* have also been previously used in algae to improve strains in terms of lipid content (Hsieh et al., 2012; Yao et al., 2014; Gomma et al., 2015) and increasing photosynthesis (Atsumi et al., 2009; Liang and Lindblad, 2017; Yang et al., 2017). These data indicate that overexpression of *CvarLOG1* had enhanced the expression of photosynthesis-related genes, which positively regulated chloroplast function, which ultimately led to an increase in biomass.

In the downregulated genes, some of the major perturbed pathways were membrane trafficking (14), cytoskeleton proteins (6), transporters (6), mitochondrial biogenesis (6), and Ubiquitin system (7) (Figures 7C,E and Supplementary Table 4, certain genes belong to more than one pathway). In *LOG* OX1 cells, the highest number of downregulated genes belonged to the membrane trafficking pathway (14) (see Supplementary Table 4). It is known that membrane trafficking is essential for plants undergoing cell cycle and is active all through the cell cycle, interphase, mitosis, and cytokinesis (Bednarek and Falbel, 2002). According to the cell cycle analysis of 14 dpi WT vs. *LOG* OX1, a higher proportion of WT cells were seen entering the mitosis stage than *LOG* OX1 cells. Therefore, the *LOG* OX1 cells had a prominent interphase stage. Previously, a difference has been detected in the membrane trafficking between cells in the interphase and mitosis (Nebenfuhr et al., 2000). Thus, this may be the reason for the downregulation of membrane trafficking genes in *LOG* OX1. Positive regulators of mitosis such as *KATANIN*, *RME1*, and *DYNEIN* (Toone et al., 1995; Niclas et al., 1996; McNally et al., 2006), also involved in the membrane trafficking pathway, are downregulated in *LOG* OX1, thus providing evidence for an association between membrane trafficking and cell cycle (Supplementary Table 4).

Other than these pathways, three genes related to the lipid pathway were downregulated, of which two were desaturases: *OMEGA-3-FATTY ACID DESATURASE* (*FAD3*) and Δ-*12 FATTY ACID DESATURASE* (*FAD2*) (Figure 7E). Downregulation of *FAD3* in *Glycine max* helps improve the quality of oil due to increased levels of linoleic acid (Flores et al., 2008). In *Jatropha*, the silencing of FAD2 aids in improving oil quality due to an increased percentage of oleic acid (Utomo et al., 2015). Both these enzyme-genes are downregulated in *LOG* OX1. Oil extraction and fatty acid profile may reveal whether there is any change in the oil quality of *LOG* OX1.

mtKAS is an enzyme involved in fatty acid synthesis in the mitochondrion (Olsen et al., 2004; Yasuno et al., 2004), which is downregulated in *LOG* OX1 (Figure 7E). Six genes involved in mitochondrial biogenesis are also downregulated in *LOG* OX1 (see Supplementary Table 4). There is evidence that mitochondrial biogenesis is induced in the M phase of the cell cycle (Shiota et al., 2015). Since there was a higher population of WT cells (14 dpi) that had already started entry into the G2/M phase, the mitochondrial biogenesis may be more elevated in WT in comparison to *LOG* OX1.

Additionally, two circadian cycle-related genes were also downregulated; C1 subunit of *CHLAMY1* and the other, a kinase *AUREOCHROME* (Figure 7E). In *Chlorella*, the G1/S phase occurs during the light cycle, whereas the G2/M phase occurs during the dark cycle. Toward the end of the light cycle, cells begin to transit toward the G2/M phase (Bisova and Zachleder, 2014). This shows that the algal cell cycle is regulated by the circadian clock (Titlyanov et al., 1996; Miyagishima et al., 2014). AUREOCHROME is a BL receptor with a LOV and bZIP domain, first discovered in stramenophile algae with a role in photo-morphogenesis (Takahashi et al., 2007). In diatoms, AUREO1a may be involved in light-dependent cell cycle activation (Huysman et al., 2013). Hence, downregulation of this gene in *LOG* OX1 indicates that there is a disruption of the circadian cycle that may have affected the cell cycle, but whether this is a direct or indirect consequence of LOG overexpression remains unknown.

Hence, RNA-Seq identified major upregulated pathways related to ribosome biogenesis, DNA replication, and photosynthesis owing to overexpression of CvarLOG1, thus indicating toward the likely existence of elevated levels of cytokinin in *LOG* OX1. Among the downregulated pathways identified, membrane trafficking is noteworthy due to the links it has with the cell cycle.

## Conclusion

Taken together, it can be concluded that the direct activation of cytokinins is functional in *Chlorella variabilis* and remains conserved from algae to flowering plants. *CvarLOG1* has a role in cell division, and cell growth of the alga and overexpression of *LOG* led to elongating the cell cycle, thereby lengthening the log phase and possibly the stationary phase as well. This created a stay-green phenotype and had a positive influence on the chloroplast function, thus leading to an increase in biomass. Since the increase in biomass and lipid yield was not comparable, further work will be required to develop a method to increase the lipid yield by altering the growth conditions. Thus, this study gives a positive prospect for the use of phytohormone-related genes in improving algal feedstock for biofuel production.

## Data Availability Statement

The original contributions presented in the study are included in the article/Supplementary Material, further inquiries can be directed to the corresponding author.

## Author Contributions

SN acquired the funding, planned the work, carried out the experiments, and wrote the manuscript.

## Conflict of Interest

The author declares that the research was conducted in the absence of any commercial or financial relationships that could be construed as a potential conflict of interest.

## Footnotes

1 http://www.megasoftware.net

2 http://www.bioinformatics.babraham.ac.uk/projects/fastqc/

3 https://www.encodeproject.org/software/trim_galore

4 https://www.ncbi.nlm.nih.gov/genome/?term=Chlorella+variabilis+NC64A

## Supplementary Material

Supplementary Figure 1 Alignment of LOG proteins from different seed and non-seed plants.

Supplementary Figure 2 Conserved domains of LOG proteins from non-seed and seed plants.

Supplementary Figure 3 SDS-PAGE of purified CvarLOG1 recombinant protein.

Supplementary Figure 4 Wild type growth curve from 1 to 29 dpi (Optical Density at 750 nm).

Supplementary Figure 5 Photograph of 45 dpi old culture of *LOG* OX1 versus wild type.

Supplementary Table 1 Accessions of LOG proteins from different seed and non-seed plants used for phylogenetic analysis.

Supplementary Table 2 List of differentially expressed genes in *LOG* OX1 versus wild type by RNA-Seq, 1.5-fold change, adjusted *p*-value (*q*-values) ≤ 0.05.

Supplementary Table 3 Major upregulated pathways in *LOG* OX1 as determined by RNA-Seq, 1.5-fold change, adjusted *p*-value (*q*-values) ≤ 0.05.

Supplementary Table 4 Major downregulated pathways in *LOG* OX1 as determined by RNA-Seq, 1.5-fold change, adjusted *p*-value (*q*-values) ≤ 0.05.

Supplementary Table 5 Primers used in this study.
