# Supplementary material for: Exploring the Role of a Cytokinin-Activating Enzyme LONELY GUY in Unicellular Microalga Chlorella variabilis
Source: Front Plant Sci. 2021 Jan 29;11:611871. doi: 10.3389/fpls.2020.611871 (PMC7891180; doi:10.3389/fpls.2020.611871)
Supplement: Supplementary Figure 1 — Alignment of LOG proteins from different seed and non-seed plants. [file Data_Sheet_1.PDF]

Supplementary Figures

Figure S1

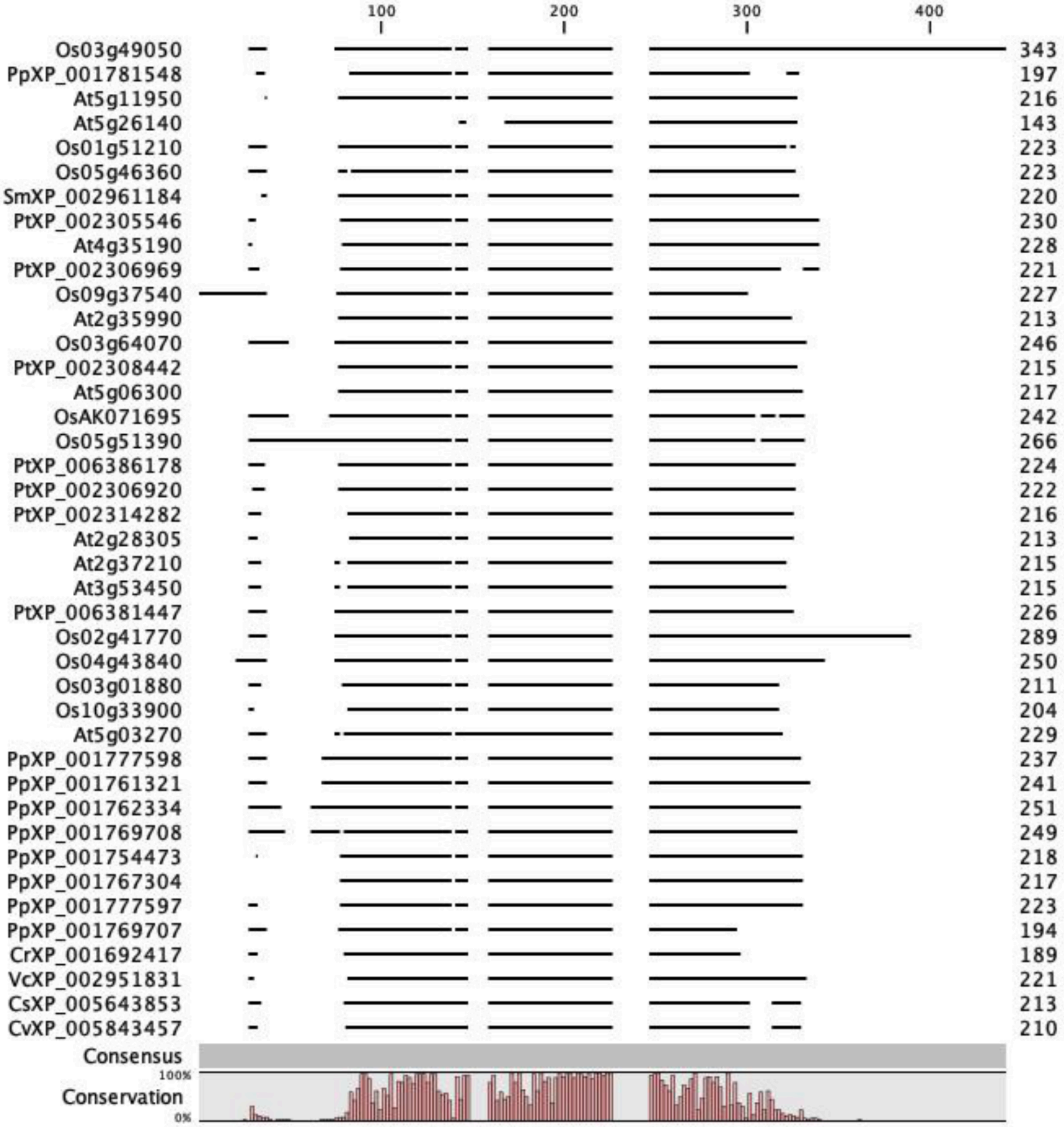

Fig. S1. Alignment of LOG proteins from different seed and non-seed plants

Figure S2

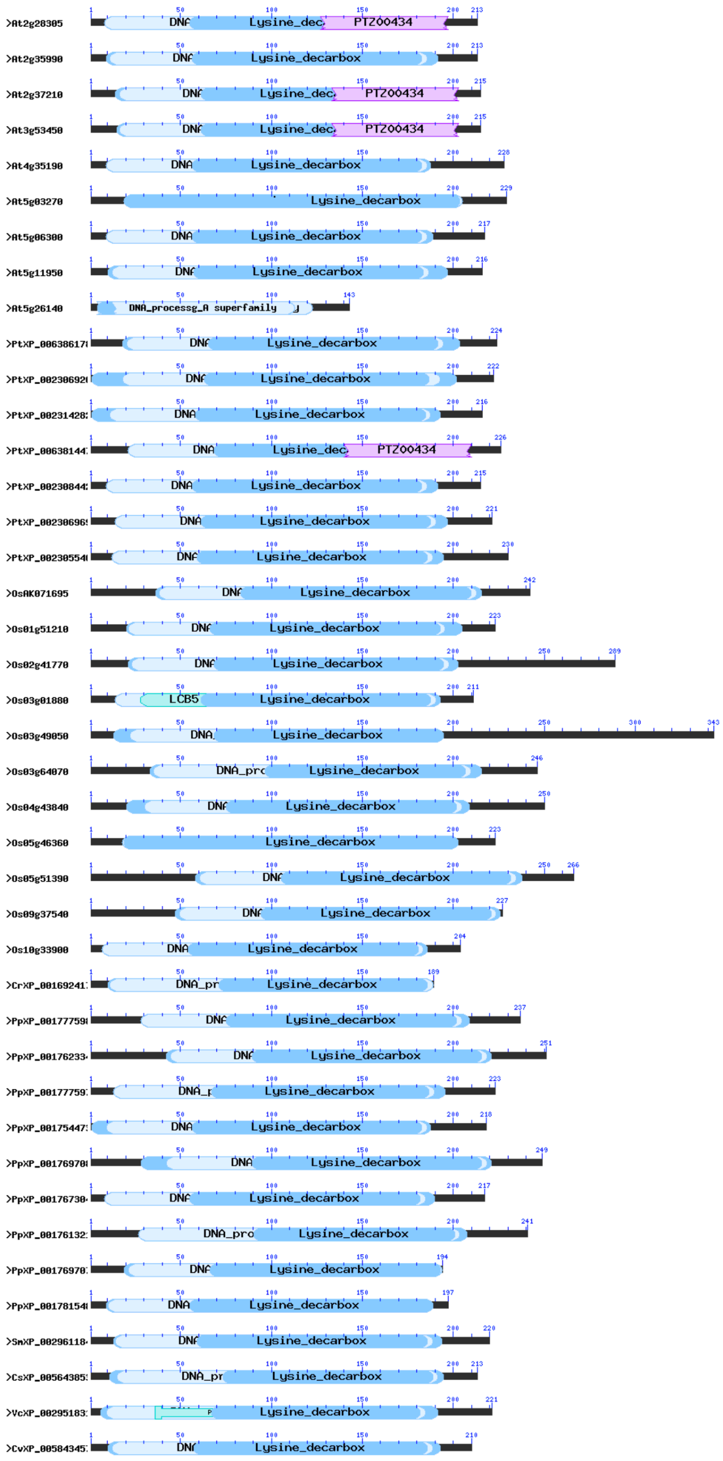

Fig. S2. Conserved domains of LOG proteins from non-seed and seed plants.

Figure S3

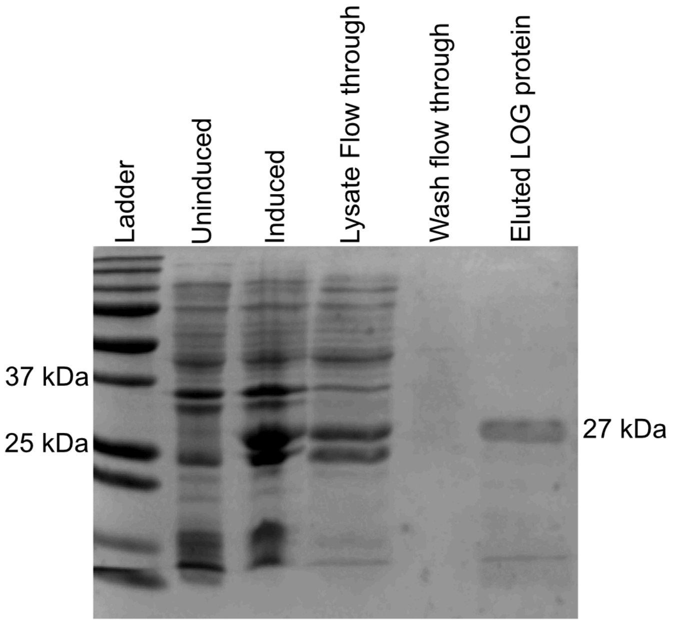

Fig. S3.SDS-PAGE of purified CvarLOG1 recombinant protein.

Figure S4

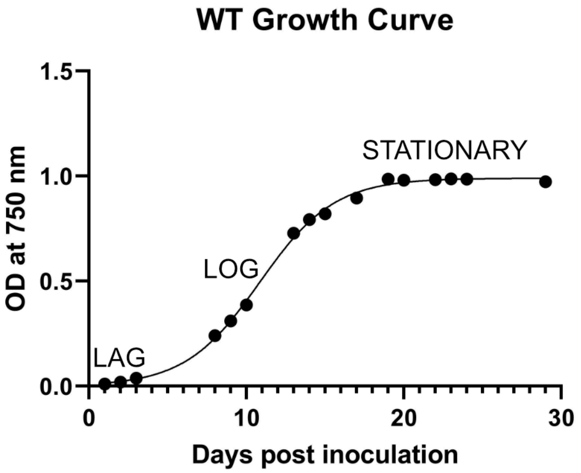

Fig. S4. Wild type growth curve from 1 dpi to 29 dpi (Optical Density at 750 nm)

Figure S5

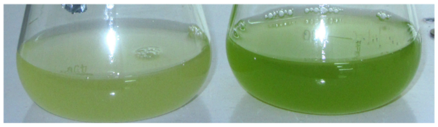

Fig. S5. Photograph of 45 dpi old culture of *LOG* OX versus wild type
